# The American Dental Association

**Published:** 1867-04

**Authors:** 


### THE AMERICAN DENTAL ASSOCIATION.

The American Dental Association, at a meeting held in Boston, beginning July 31st, 1866, passed the following resolution :

*Resolved*, 1st. That a Committee of three be appointed to draft suitable suggestions upon the subject of accepting students, and that such suggestions be printed in circular form for the consideration of every Dental practitioner in the United States.

2d. That the expense of such printing and distribution be borne by the Association.

The undersigned, the Committee appointed according to the provisions of the above resolution, are fully persuaded that the time has now arrived when every Dental practitioner in the country can and should lend his aid in elevating the status of the profession, to the end that those who are soon to fill our places may be prepared, in a greater degree, to fulfill the reasonable expectations of the public and hold Dentistry in its proper rank among the learned professions.

Our Dental Colleges have done much, and will doubtless do more, but there is a work for the private instructor to accomplish, that students may be better qualified to enter such collegiate institutions and graduate with credit to themselves and honor to the profession. It is not only essential that Dental Colleges exist, but they should be furnished with properly qualified pupils to ensure that success and usefulness contemplated in their foundation.

Relying, then, upon the generous co-operation of our professional brethren, we respectfully submit the following "suggestions" as a basis in "accepting Dental Students:"

1. He must possess a good moral character, and at least a good English education.

2. He must be required to apply himself diligently for three years, including two full courses of lectures in some Dental College, to the following studies, viz:

*First Year.*—Anatomy, Histology and Physiology.

*Second Year.*—Pathology, Chemistry, Metallurgy and Mechanical Dentistry.

*Third Year.*—Operative Dentistry, Special Pathology, Dental Medicine and Microscopy.

We further suggest that the instructor examine his pupil in his studies at least twice in every week, and as much oftener as may be convenient—not, however, including his lecture terms—and should the student, after sufficient trial, fail to exhibit the necessary talent for our specialty, he should be kindly apprised of the fact, and advised to seek other fields of usefulness.

A. LAWRENCE, *Lowell, Mass.*
C. P. FITCH, *New York City.*
J. TAFT, *Cincinnati, Ohio.*

